# Aumento da Rigidez Arterial Pulmonar e Comprometimento do Acoplamento Ventrículo Direito-Artéria Pulmonar na SOP

**DOI:** 10.36660/abc.20190762

**Published:** 2021-04-08

**Authors:** Ozge Ozcan Abacioglu, Erdinc Gulumsek, Hilmi Sumbul, Mehmet Kaplan, Fethi Yavuz

**Affiliations:** 1 Health Sciences University Adana Research and Training Hospital Department of Cardiology Adana Turquia Health Sciences University, Adana Research and Training Hospital, Department of Cardiology, Adana - Turquia; 2 Health Sciences University Adana Research and Training Hospital Department of Internal Medicine Adana Turquia Health Sciences University, Adana Research and Training Hospital, Department of Internal Medicine, Adana - Turquia

**Keywords:** Doenças do Sistema Endócrino, Rigidez Arterial, Infertilidade Feminina, Obesidade, Dispneia, Hipertensão Pulmonar

## Abstract

**Fundamento:**

A síndrome dos ovários policísticos (SOP) é a doença endócrino-metabólica mais comum em mulheres em idade reprodutiva, e ocorre em uma a cada 10 mulheres. A doença inclui irregularidade menstrual e excesso de hormônios masculinos e é a causa mais comum de infertilidade em mulheres. A dispneia é um sintoma frequente e muitas vezes acredita-se que seja decorrente da obesidade, mas não se sabe se é decorrente de disfunção cardíaca.

**Objetivo:**

Avaliar o acoplamento ventrículo-arterial (VDAP) e a rigidez arterial pulmonar em pacientes com SOP.

**Métodos:**

Foram incluídos 44 pacientes com SOP e 60 controles; amostras de sangue venoso foram coletadas para exames laboratoriais e ecocardiograma transtorácico 2-D, Modo-M e com Doppler tecidual foram realizados em todos os participantes. Um valor de p<0,05 foi considerado estatisticamente significativo.

**Resultados:**

Quando comparadas ao grupo controle, as pacientes com SOP apresentaram valores maiores de rigidez da artéria pulmonar (p = 0,001), que se correlacionaram positivamente com o índice HOMA-IR (r = 0,545 e p <0,001). O acoplamento VDAP também estava comprometido em 34% dos pacientes do estudo.

**Conclusão:**

A rigidez da artéria pulmonar está aumentada e o acoplamento VDAP está comprometido em pacientes com SOP. (Arq Bras Cardiol. 2021; 116(4):806-811)

## Introdução

A síndrome do ovário policístico (SOP) é considerada uma doença multissistêmica, reprodutiva e metabólica. É o distúrbio endocrinológico mais comum em mulheres em idade reprodutiva e sua prevalência varia entre 6 a 15% de acordo com diferentes critérios diagnósticos. A fim de esclarecer os critérios diagnósticos da SOP, três consensos principais foram estabelecidos até o momento (*National Institutes of Health* – NIH, *Rotterdam and Androgen Excess Society*). A presença de ovários policísticos, irregularidade menstrual, hirsutismo, obesidade e resistência à insulina (RI) contribuem para o quadro clínico da SOP.^1^ Mulheres com SOP apresentam perfil de risco cardiovascular adverso, incluindo dislipidemia, hipertensão e também disfunção endotelial e calcificação das artérias coronárias.^2^^,^^3^ Estudos recentes demonstraram que o comprometimento assintomático da função do VE em mulheres jovens está associado à obesidade e RI, em vez dos distúrbios de hormônios sexuais associados à SOP e, em outro estudo, verificou-se que a massa do VE foi maior em pacientes com SOP.^2^^–^^4^

A rigidez arterial pulmonar (RAP) foi desenvolvida como um parâmetro ecocardiográfico Doppler relativamente novo para avaliar a vasculatura arterial pulmonar e sua mecânica.^5^^,^^6^ Sua associação com a função do ventrículo direito (VD) e a capacidade de predizer a capacidade funcional na hipertensão pulmonar foi demonstrada. A RAP está aumentada no início do desenvolvimento da hipertensão pulmonar e, portanto, estudos sugerem que esse biomarcador pode ser utilizado para detecção precoce da doença.

O acoplamento ventrículo direito-artéria pulmonar é um indicador de complacência arterial pulmonar e seu comprometimento é resultado da redução da complacência da artéria pulmonar.^7^ Estudos sugeriram que a diminuição da complacência desempenha um papel crítico na patogênese da hipertensão arterial pulmonar (HAP), de modo que o acoplamento VDAP é clinicamente importante, devido à sua associação com aumento da mortalidade em pacientes com HAP.

O objetivo deste estudo foi investigar a rigidez da artéria pulmonar e o acoplamento VDAP em pacientes com SOP.

## Métodos

### População de estudo

A coorte do estudo consistiu em 104 pacientes recrutados da *Internal Diseases Policlinic* do *Adana City Education and Research Hospital* entre março de 2019 e setembro de 2019. Foram obtidos dados sobre características demográficas, histórico médico e uso de medicamentos, sendo excluídos os pacientes que apresentavam doença arterial coronariana, hipertensão, diabetes mellitus, doença cardíaca valvar em vez de disfunção diastólica leve, diagnóstico ou achados clínicos (ronco, sonolência diurna excessiva ou apneia testemunhada) de síndrome da apneia obstrutiva do sono, hipertensão arterial pulmonar, doença respiratória, disfunção sistólica do ventrículo direito e imagens ecocardiográficas de má qualidade. O Índice de Massa Corporal foi calculado como o peso em quilogramas dividido pelo quadrado da altura em metros. Critérios do NIH: foram usados para o diagnóstico: hiperandrogenismo clínico e/ou bioquímico, disfunção ovariana (oligoanovulação e/ou ovários policísticos) e exclusão de outras causas, como síndrome de Cushing, tumores etc. A população do estudo era assintomática, com 77% de hirsutismo, 32% de irregularidade menstrual, 6% de acne, 6% de infertilidade e 6% de obesidade. O escore da insuficiência cardíaca com fração de ejeção preservada (ICFEp) foi 0 ou 1 em 91% dos participantes e a possibilidade de insuficiência cardíaca foi baixa nos grupos. Treze (29%) pacientes com SOP recebiam diferentes tratamentos. Apenas uma delas estava utilizando metformina. A duração média da doença foi de 31 meses. O grupo controle era constituído de pacientes atendidas na policlínica com sintomas semelhantes, mas que não preenchiam os critérios para SOP, sendo 66% com irregularidade menstrual, 20% com acne e 14% com infertilidade. As causas desses sintomas eram a dieta, distúrbios hormonais e estresse; restrição de gordura na dieta, tratamento da ansiedade e medicamentos para diminuir os níveis de prolactina foram administrados, com diminuição dos sintomas. O *Adana City Education and Research Hospital* aprovou o protocolo do estudo e o mesmo foi realizado de acordo com os princípios da Declaração de Helsinque.

### Ecocardiografia

Uma avaliação foi realizada utilizando ecocardiografia transtorácica (ETT) completa com um equipamento de ultrassonografia comercialmente disponível de acordo com as recomendações da *American Society of Echocardiography.*^8^ Os exames de ETT incluíram avaliações em modo M, bidimensional, avaliações de fluxo com Doppler e Doppler tecidual e pulsado. Foram determinadas a fração de ejeção do VE (FEVE) e a espessura da parede posterior (EPP) e do septo interventricular (ESIV). Foram determinadas a velocidade diastólica inicial e tardia da válvula tricúspide, a pressão sistólica da artéria pulmonar (PSAP) e a velocidade máxima do fluxo pulmonar. A excursão sistólica do plano do anel tricúspide (TAPSE, do inglês *tricuspid annular plane systolic excursio*n), uma medida do desempenho do VD, foi realizada a partir da análise do Modo M na visão apical de quatro câmaras com foco no VD. O tempo de aceleração da artéria pulmonar (PAAT, do inglês *pulmonary artery acceleration time*) foi obtido a partir da visão do eixo longo paraesternal do fluxo de saída do VD ao nível da válvula pulmonar, utilizando um protocolo publicado para aquisição de imagens de PAAT.

A rigidez da artéria pulmonar foi avaliada na visão do eixo curto paraesternal com Doppler pulsado e calculada de acordo com a seguinte fórmula: a razão entre o deslocamento máximo da velocidade do fluxo pulmonar e o tempo de aceleração do fluxo pulmonar.^9^

A relação entre a contratilidade do VD e a pós-carga do VD é frequentemente chamada de acoplamento VDAP. A contratilidade refere-se à função cardíaca intrínseca ou independente da carga, enquanto a pós-carga refere-se à oposição à ejeção ventricular. O acoplamento VDAP foi calculado de acordo com a seguinte fórmula: TAPSE/PSAP e quando um valor <1,6 era obtido, isso era caracterizado como acoplamento deficiente.^10^

As medidas ecocardiográficas foram realizadas por dois ecocardiografistas, com mascaramento. A média das medidas foram calculadas.

### Análise laboratorial

As análises laboratoriais incluem hemograma completo de rotina, níveis bioquímicos e de insulina de ambos os grupos, de estudo e controle. Os níveis séricos de lipoproteína de baixa densidade-colesterol (LDL-c), lipoproteína de alta densidade-colesterol (HDL-c) e triglicérides (TG) foram medidos utilizando azul de xilidina com um método colorimétrico de ponto final. O modelo de avaliação da homeostase da resistência à insulina (HOMA-IR) foi calculado utilizando o teste de glicemia em jejum, com exame de insulina em jejum de pelo menos 8-10 horas e de acordo com a seguinte fórmula: nível de glicose em jejum (mg/dL) x nível de insulina em jejum (uIU/mL)/405. Um escore HOMA ≥2,5 foi considerado como positivo para a resistência à insulina.

### Análise estatística

Todas as análises estatísticas foram realizadas utilizando o software SPSS 17 (SPSS, Inc., Chicago, Illinois, EUA). As variáveis do estudo foram analisadas por métodos analíticos (teste de Kolmogorov-Smirnov) para determinar a distribuição normal e foram expressas como média ± desvio padrão (média ± DP) ou números e porcentagens. O teste U de Mann-Whitney foi utilizado para a comparação de 2 grupos com uma distribuição não-normal das variáveis e o teste do qui-quadrado para a comparação dos dados qualitativos. As comparações das variáveis contínuas entre os grupos foram realizadas pelo teste *t* para amostras independentes, quando apropriado, e as associações entre as variáveis foram realizadas pela correlação produto-momento de Pearson. Um valor de p bicaudal menor que 0,05 foi considerado significativo. A reprodutibilidade inter-observador foi medida com o coeficiente de correlação tau-b de Kendall.

## Resultados

O grupo com SOP tinha média de idade de 22 ± 5 anos, enquanto no grupo controle a idade média era de 24 ± 5 anos. A idade e o índice de massa corporal foram estatisticamente semelhantes nos grupos (p = 0,329 e 0,210, respectivamente). As características demográficas basais e os parâmetros laboratoriais dos grupos de estudo são mostrados na Tabela 1.

**Tabela 1:** Características demográficas basais e parâmetros laboratoriais dos grupos e análise estatística

	Grupo SOP n=44 (média ± DP)	Grupo Controle n=60 (média ± DP)	p-valor
Idade, anos	22 ± 5	24 ± 5	0,210
IMC, kg/ m^2^	24,86 ± 2,74	24,26 ± 2,25	0,329
Glicose (mg/ dL)	96,45 ± 12,52	90,16 ± 1,48	0,279
Ureia (mg/dL)	20,22 ± 5,53	23,38 ± 3,96	0,233
Sódio (mmol/L)	139,25 ± 1,72	137,60 ± 0,52	0,114
Potássio (mmol/L)	4,43 ± 0,29	4,33 ± 0,14	0,568
Cálcio (mg/dL)	9,75 ± 0,35	9,62 ± 0,60	0,473
AST (u /L)	20,72 ± 5,06	19,88 ± 5,45	0,735
ALT (u /L)	16,90 ± 9,10	13,02 ± 2,01	0,354
LDL (mg /dL)	119,25 ± 22,81	111,16 ± 32,26	0,580
HDL (mg dL)	46,13 ± 13,28	42,30 ± 15,46	0,317
Triglicérides (mg /dL)	106,30 ± 78,40	91,66 ± 50,63	0,757
CL (10^3^/µL)	7,60 ± 1,76	8,44 ± 2,79	0,318
HGB (g/dL)	12,90 ± 0,81	11,85 ± 2,10	0,238
PLT (10^3^/µL)	277,90 ± 69,23	272,85 ± 33,25	0,853
HOMA-IR	3,12 ± 2,00	2,16 ±, 52	0,023

IMC: índice de massa corporal; AST: aspartato aminotransferase; ALT: alanina aminotransferase; LDL: lipoproteína de baixa densidade, HDL: lipoproteína de alta densidade, CL: contagem leucocitária, HGB: hemoglobina, PLT: plaquetas, HOMA-IR: modelo de avaliação da homeostase da resistência à insulina.

As características da ecocardiografia ventricular esquerda e direita são apresentadas na Tabela 2. A fração de ejeção do VE, espessura do septo interventricular e da parede posterior, velocidades diastólicas da válvula tricúspide inicial (E) e tardia (A), PSAP e as velocidades máximas da artéria pulmonar foram semelhantes entre os grupos. A TAPSE foi menor e o tempo de aceleração do fluxo arterial pulmonar foi encurtado no grupo de estudo, com diferença estatisticamente significativa (p <0,001 e p = 0,001, respectivamente).

**Tabela 2:** Características ecocardiográficas dos ventrículos esquerdo e direito dos grupos de estudo e controle e análise estatística

	Grupo SOP n=44 (média ± DP)	Grupo Controle n=60 (média ± DP)	p-valor
FEVE (%)	61,45 ± 5,76	61,00 ± 5,32	0,810
ESIV (mm)	8,85 ± 1,07	8,98 ± 1,24	0,633
EPP (mm)	8,34 ± 1,06	8,63 ± 1,47	0,329
E/E’	10,33 ± 1,57	10,39 ± 1,76	0,896
Velocidade E da tricúspide (cm/s)	80,25 ± 12,73	75,81 ± 12,20	0,140
Velocidade A da tricúspide (cm/s)	56,05 ± 8,33	56,25 ± 9,76	0,924
PSAP (mmHg)	19,04 ± 2,54	18,04 ± 1,74	0,064
TA (ms)	159,35 ± 24,08	179,17 ± 22,36	0,001
Velocidade pulmonar máxima	87,38 ± 12,49	84,79 ± 6,21	0,299
TAPSE (cm)	2,18 ± 0,30	2,58 ± 0,25	<0,001
RAP	5,58 ± 1,05	4,80 ± 0,78	0,001
Acoplamento VDAP	1,09 ± 0,23	1,63 ± 0,31	<0,001

SOP: síndrome dos ovários policísticos; FEVE: fração de ejeção do ventrículo esquerdo; ESIV: septo interventricular; EPP: espessura da parede posterior; PSAP: pressão sistólica da artéria pulmonar; TAPSE: do inglês tricuspid annular plane systolic excursion; RAP: rigidez arterial pulmonar; VDAP: acoplamento ventrículo-arterial. TA: tempo de aceleração.

**Figura 1:**
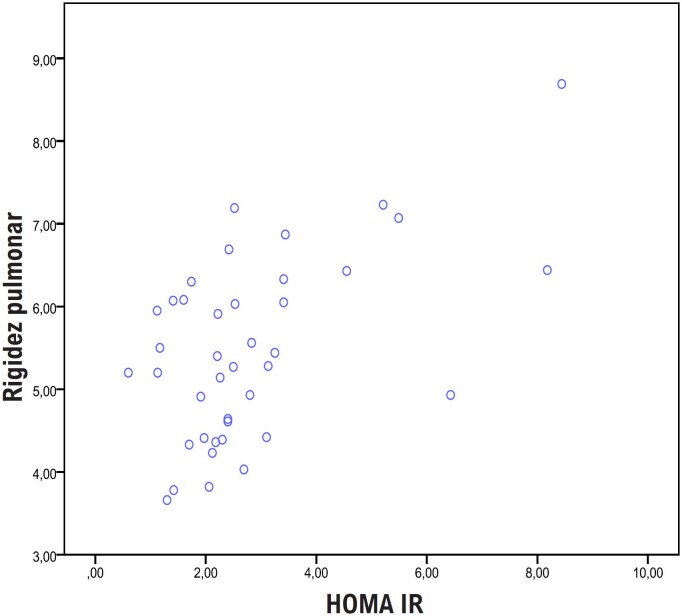
Correlação entre HOMA-IR e RAP.

Os níveis de rigidez da artéria pulmonar (RAP) foram mais elevados no grupo SOP e a RAP apresentou correlação significativamente positiva com o HOMA-IR (r = 0,545 e p <0,001) (Tabela 2 e Figura 1). Seis pacientes (46%) com resistência à insulina apresentaram valores de RAP mais elevados do que os controles. A análise de subgrupo das pacientes do estudo que receberam tratamento e aquelas que não receberam tratamento mostrou que a rigidez da artéria pulmonar foi maior no grupo sem tratamento (RAP = 5,15 ± 0,99 e 5,75 ± 1,02 respectivamente), mas a diferença não foi estatisticamente significativa (p = 0,084).

O acoplamento VDAP estava comprometido em 15 pacientes (34%) do grupo de estudo, com níveis médios de 1,09 ± 0,23 e o valor de *p* foi significativo entre os dois grupos (p <0,001). Treze dessas 15 pacientes não estavam recebendo nenhum tratamento e a diferença em termos de valores de acoplamento VDAP entre os subgrupos, tratados ou não tratados, também foi estatisticamente significativa. O valores do acoplamento VDAP foram (média ± DP) 1,20 ± 0,22 para o grupo tratado e 1,05 ± 0,22 para o grupo não tratado. O valor de p foi de 0,048.

O valor do coeficiente de correlação tau-b de Kendall foi 0,961 para a RAP e 0,790 para o acoplamento VDAP.

## Discussão

É bem conhecido que o risco de doenças cardiovasculares é elevado em pacientes com SOP, devido ao aumento da resistência à insulina e diminuição da tolerância à glicose. Achados relatados anteriormente sobre a resistência à insulina e seu metabolismo fornecem novas pistas no tratamento da SOP e complicações relacionadas.^11^

As manifestações clínicas da resistência à insulina são: hipertensão (HT), dislipidemia e diabetes mellitus tipo 2 (DM-2). Os efeitos assintomáticos são disfunção endotelial, estado pró-coagulante, condição pró-inflamatória e proliferação de células de músculo liso. Ergun et al, descobriram que pacientes com síndrome metabólica tinham valores de rigidez aórtica maiores do que os controles. O mecanismo pelo qual a resistência à insulina aumenta a rigidez pode ser explicado por seus efeitos assintomáticos.^12^

Wang et.al.,^4^ relataram no estudo CARDIA, realizado com mulheres, que a síndrome dos ovários policísticos está associada a um índice de massa ventricular esquerdo mais alto e, em outro estudo, foram observadas anormalidades distintas nas características cardiovasculares e metabólicas na SOP em idade precoce.^13^^–^^14^ Essas diferenças são refletidas por uma pressão de pulso aumentada e uma pressão diastólica final do ventrículo esquerdo mais alta, mas uma imagem Doppler tecidual de mais baixa qualidade da parede direita na sístole. Os resultados podem indicar que as mulheres com SOP já apresentam disfunção arterial sutil, o que pode levar à aterosclerose mais tarde.

A rigidez arterial pulmonar e a hemodinâmica anormal de fluxo na hipertensão arterial pulmonar estão fortemente associadas à pós-carga ventricular direita elevada e à gravidade da doença e desfechos clínicos desfavoráveis em adultos com HAP.^15^^–^^17^ O acoplamento VDAP pode descrever a compensação do VD na hipertensão pulmonar e também em doenças cardíacas esquerdas, e sua importância tem aumentado juntamente com o reconhecimento crescente do papel central que o VD desempenha em muitas condições cardiopulmonares.^18^^–^^20^

Descobrimos que a rigidez da artéria pulmonar, um indicador da vasculatura da artéria pulmonar, estava aumentada na SOP e foi associada a níveis mais elevados do HOMA-IR. O acoplamento VDAP, um indicador da complacência arterial pulmonar que tem papel importante na patogênese da hipertensão arterial pulmonar, está prejudicado nesse grupo de pacientes. Este estudo é o primeiro a examinar a rigidez da artéria pulmonar e o acoplamento VDAP em pacientes com SOP.

Considerando todas essas complicações e eventos, foi demonstrado em muitos estudos anteriores e meta-análises que a patologia subjacente é a resistência à insulina. Embora os estudos sobre ventrículo esquerdo e doença arterial coronariana sejam a maioria, a hipertensão pulmonar e a disfunção ventricular direita têm um papel significativo na mortalidade e estabelecem sérias limitações para a qualidade de vida do paciente. As pacientes com SOP devem ser informadas sobre o risco cardíaco e o exames cardíacos de rotina deve ser recomendados.

### Limitações

Nosso estudo apresentou algumas limitações. Em primeiro lugar, foi um estudo de centro único com poucos participantes. Outra limitação do estudo atual foi o curto período de seguimento. Além disso, a avaliação da resistência à insulina foi baseada apenas no índice HOMA-IR. Outras investigações com maior duração e com grupos maiores são necessárias para examinar a sustentabilidade dos resultados.

## Conclusão

Em resumo, este estudo é o primeiro a fornecer dados preliminares de que pacientes com SOP têm aumento da rigidez da artéria pulmonar e acoplamento VDAP prejudicado.
