# Supplementary material for: Aumento da Rigidez Arterial Pulmonar e Comprometimento do Acoplamento Ventrículo Direito-Artéria Pulmonar na SOP
Source: Arq Bras Cardiol. 2021 Apr 8;116(4):806–11. [Article in Portuguese] doi: 10.36660/abc.20190762 (PMC8121413; doi:10.36660/abc.20190762)
Supplement: Translation

# Translation: Increased Pulmonary Arterial Stiffness and Impaired Right Ventricle-Pulmonary Artery Coupling In PCOS

**Authors:** Ozge Ozcan Abacioglu, Erdinc Gulumsek, Hilmi Sumbul, Mehmet Kaplan, Fethi Yavuz

**Affiliations:** 1 Adana Turkey Health Sciences University, Adana Research and Training Hospital, Department of Cardiology, Adana - Turkey; 2 Adana Turkey Health Sciences University, Adana Research and Training Hospital, Department of Internal Medicine, Adana - Turkey

**Keywords:** Diseases of the Endocrine System, Arterial Stiffness, Female infertility, Obesity, Dyspnea, Pulmonary hypertension

## Abstract

**Background:**

Polycystic ovary syndrome (PCOS) is the most common endocrine-metabolic disease in women in reproductive age, and occurs in one of 10 women. The disease includes menstrual irregularity and excess of male hormones and is the most common cause of female infertility. Dyspnea is a frequent symptom and is often thought to be due to obesity, and whether it is due to cardiac dysfunction is unknown.

**Objective:**

To evaluate right ventricle-pulmonary artery (RV-PA) coupling and pulmonary arterial stiffness in patients with PCOS.

**Methods:**

44 PCOS patients and 60 controls were included; venous blood samples were taken for laboratory tests and 2-D, m-mode and tissue doppler transthoracic echocardiography were performed for all the participants. P<0,05 was considered as statistically significant.

**Results:**

When compared to the control group, PCOS patients had higher pulmonary artery stiffness values (p=0,001), which were positively correlated with HOMA-IR (r=0,545 and p<0,001). RV-PA coupling was also impaired in 34% of the study patients.

**Conclusion:**

Pulmonary artery stiffness is increased and RV-PA coupling is impaired in patients with PCOS. (Arq Bras Cardiol. 2021; 116(4):806-811)

## Introduction

Polycystic ovary syndrome (PCOS) is considered a multisystemic, reproductive and metabolic disease. It is the most common endocrinological disorder in women of reproductive age and its prevalence varies between 6-15% according to different diagnostic criteria. In order to clarify the diagnostic criteria of PCOS, three major consensuses have been established to date (National Institute of Health- NIH, Rotterdam and Androgen Excess Society). The presence of polycystic ovaries, menstrual irregularity, hirsutism, obesity and insulin resistance (IR) contribute to the clinical presentation of PCOS.^1^ Women with PCOS have an adverse cardiovascular risk profile including dyslipidemia, hypertension and also endothelial dysfunction and coronary artery calcification.^2^^,^^3^ Recent studies have shown that asymptomatic impairment of left ventricular (LV) function in young women is associated with obesity and IR rather than the sex hormone disturbances associated with PCO and in another study, LV mass was found to be higher in PCOS patients.^2^^–^^4^

Pulmonary artery stiffness (PAS) has been developed as a relatively new Doppler echocardiographic parameter to evaluate the pulmonary artery vasculature and mechanics.^5^^,^^6^ Its association with right ventricular (RV) function and ability to predict functional capacity in pulmonary hypertension has been demonstrated. PAS is increased early in pulmonary hypertension development, so studies suggest that this biomarker may be used for early disease detection.

The right ventricle - pulmonary artery coupling is an indicator of pulmonary arterial compliance and its impairment is a result of reduced pulmonary artery compliance.^7^ Studies suggested that decreased compliance plays a critical role in the pathogenesis of pulmonary artery hypertension (PAH) so that RV-PA coupling is clinically important, because of its association with increased mortality in patients with PAH.

The aim of this study was to investigate pulmonary artery stiffness and RV-PA coupling in patients with PCOS.

## Methods

### Study population

The study cohort consisted of 104 patients recruited from the Internal Diseases policlinic of Adana City Education and Research Hospital between March 2019 and September 2019. Data on demographic characteristics, medical history and medication use were obtained and patients with coronary artery disease, hypertension, diabetes mellitus, valvular heart disease rather than mild diastolic dysfunction, diagnosis or clinical findings (snoring, excessive daytime sleepiness or witnessed apnea) of obstructive sleep apnea syndrome, pulmonary artery hypertension, respiratory disease, right ventricular systolic dysfunction and poor echocardiographic imaging were excluded. Body mass index was calculated as the weight in kilograms divided by the squared height in meters. NIH criteria: clinical and/or biochemical hyperandrogenism, ovarian dysfunction (oligo-anovulation and /or polycystic ovaries) and exclusion of other causes such as Cushing syndrome, tumors etc., were used for diagnosis. The study population was asymptomatic and 77% presented with hirsutism, 32% with menstrual irregularity, 6% with acne, 6% with infertility and 6% with obesity. HFpEF (heart failure with preserved ejection fraction) score was 0 or 1 in 91% of the participants and the possibility of heart failure was low in the groups. 13 (29%) of PCOS patients were undergoing different treatments. Only one of them was using metformin. Mean disease duration was 31 months. The control group consisted of patients admitted to the polyclinic with similar symptoms but who did not meet the criteria for PCOS, of which 66% with menstrual irregularity, 20% with acne and 14% with infertility. These were due to diet, hormonal disorder and stress; fat restriction in the diet, anxiety treatment and prolactin lowering medications were administered and symptoms were relieved. The Adana City Education and Research Hospital approved the study protocol and this study was performed in accordance with the Declaration of Helsinki principles.

### Echocardiography

A complete transthoracic echocardiographic evaluation was performed using commercially available ultrasonographic equipment according to recommendations of the American Society of Echocardiography.^8^ TTE examinations included M-mode, two dimensional, Doppler flow assessments and pulsed-wave tissue Doppler imaging measurements. LV ejection fraction (LVEF), posterior wall (PW) and interventricular septal thickness (IVS) were determined. Tricuspid early and late diastolic velocities, systolic pulmonary arterial pressure, maximal pulmonary velocity were determined. Tricuspid annular plane systolic excursion (TAPSE), a measure of RV performance, was measured using m-mode analysis in the RV-focused apical four chamber view. Pulmonary artery acceleration time (PAAT), was acquired from the parasternal long axis view of the RV outflow at the level of the pulmonary valve using a published protocol for PAAT image acquisition.

Pulmonary artery stiffness was assessed in the parasternal short axis view using pulsed-wave Doppler and calculated according to the following formula: the ratio of maximum flow velocity shift of pulmonary flow to pulmonary acceleration time.^9^

The relationship between RV contractility and RV afterload is often referred to as RV-PA coupling. Contractility refers to load independent or intrinsic cardiac function, while afterload refers to the opposition to ventricular ejection. RV- PA coupling was calculated according to the following formula: TAPSE / SPAP and when a ratio <1.6 was obtained, it was characterized as impaired coupling.^10^

The echocardiographic measurements were performed by two blinded echocardiographers. The averages of measurements were calculated.

### Laboratory analysis

Laboratory analysis included routine complete blood count, biochemistry and insulin levels for both the study and control groups. Serum lipid levels of low-density lipoprotein (LDL) cholesterol, high-density lipoprotein (HDL) cholesterol, and triglycerides (TG) were measured using xylidine blue with an endpoint colorimetric method. The Homeostatic Model Assessment for Insulin Resistance (HOMA-IR) was calculated using fasting blood glucose level with fasting insulin level with at least 8-10 hours of fasting and according to following formula: fasting glucose level (mg /dL) x fasting insulin level (uIU/mL) /405. A HOMA score ≥2.5 was considered as positive insulin resistance.

### Statistical analysis

All statistical analyses were performed using SPSS 17 (SPSS, Inc., Chicago, Illinois, USA). The study variables were analyzed using analytical methods (Kolmogorov-Smirnov test) to determine normal distribution and were expressed as mean ± standard deviation (mean ± SD) or numbers and percentages. The Mann-Whitney U test was used for the comparison of 2 groups with a non-normal distribution of variables and the chi-square test was used for the comparison of qualitative data. Comparisons of the continuous variables between groups were performed using the independent samples t-test, as appropriate, and associations between variables were carried out using Pearson ‘s product moment test. A two-tailed p value of less than 0.05 was considered as significant. Interobserver reproducibility was measured with Kendall's tau b correlation coefficient.

## Results

The PCOS group's mean age was 22 ± 5 years, while the control group was 24 ± 5 years. Age and body mass index were statistically similar in the groups (p=0.329 and 0.210 respectively). The baseline demographic characteristics and laboratory parameters of the study groups are shown in Table 1.

**Table 1:** Baseline demographic features and laboratory parameters of groups, and statistical analysis

	PCOS group n=44 (mean ± SD)	Control group n=60 (mean ± SD)	p-value
Age, years	22 ± 5	24 ± 5	0.210
BMI, kg/ m^2^	24.86 ± 2.74	24.26 ± 2.25	0.329
Glucose (mg/ dL)	96.45 ± 12.52	90.16 ± 1.48	0.279
Urea (mg / dL)	20.22 ± 5.53	23.38 ± 3.96	0.233
Sodium (mmol / L)	139.25 ± 1.72	137.60 ± 0.52	0.114
Potassium (mmol /L)	4.43 ± 0.29	4.33 ± 0.14	0.568
Calcium (mg / dL)	9.75 ± 0.35	9.62 ± 0.60	0.473
AST (u /L)	20.72 ± 5.06	19.88 ± 5.45	0.735
ALT (u /L)	16.90 ± 9.10	13.02 ± 2.01	0.354
LDL (mg /dL)	119.25 ± 22.81	111.16 ± 32.26	0.580
HDL (mg dL)	46.13 ± 13.28	42.30 ± 15.46	0.317
Triglycerides (mg /dL)	106.30 ± 78.40	91.66 ± 50.63	0.757
WBC (10^3^ /µL)	7.60 ± 1.76	8.44 ± 2.79	0.318
HGB (g /dL)	12.90 ± 0.81	11.85 ± 2.10	0.238
PLT (10^3^ /µL)	277.90 ± 69.23	272.85 ± 33.25	0.853
HOMA-IR	3.12 ± 2.00	2.16 ± .52	0.023

BMI: body mass index; AST: aspartate transaminase, ALT: alanine transaminase, LDL: low-density lipoprotein, HDL: high-density lipoprotein, WBC: white blood count, HGB: hemoglobin, PLT: platelets, HOMA-IR: homeostatic model for insulin resistance.

Left and right ventricular echocardiography characteristics are shown in Table 2. LV ejection fraction, interventricular septum and posterior wall thickness, tricuspid early (E) and late (A) diastolic velocities, systolic pulmonary artery pressure (SPAP) and maximum pulmonary artery velocities were similar between groups. TAPSE was lower and pulmonary acceleration time was shortened in the study group and the difference was statistically significant (p<0.001 and p= 0.001, respectively).

**Table 2:** Left and right ventricular echocardiography characteristics of the study and control groups, and statistical analysis

	PCOS group n=44 (mean ± SD)	Control group n=60 (mean ± SD)	p-value
LVEF (%)	61.45 ± 5.76	61.00 ± 5.32	0.810
IVS (mm)	8.85 ± 1.07	8.98 ± 1.24	0.633
PW (mm)	8.34 ± 1.06	8.63 ± 1.47	0.329
E/E’	10.33 ± 1.57	10.39 ± 1.76	0.896
Tricuspid E velocity (cm/s)	80.25 ± 12.73	75.81 ± 12.20	0.140
Tricuspid A velocity (cm/s)	56.05 ± 8.33	56.25 ± 9.76	0.924
SPAP (mmHg)	19.04 ± 2.54	18.04 ± 1.74	0.064
AT (ms)	159.35 ± 24.08	179.17 ± 22.36	0.001
Maximum Pulmonary Velocity	87.38 ± 12.49	84.79 ± 6.21	0.299
TAPSE (cm)	2.18 ± 0.30	2.58 ± 0.25	<0.001
PAS	5.58 ± 1.05	4.80 ± 0.78	0.001
RV-PA coupling	1.09 ± 0.23	1.63 ± 0.31	<0.001

PCOS: polycystic ovary syndrome; LVEF: left ventricular ejection fraction; IVS: interventricular septal thickness; PW: posterior wall; SPAP: systolic pulmonary artery pressure; AT: Acceleration time; TAPSE: tricuspid annular plane systolic excursion; PAS: pulmonary artery stiffness; RV-PA: right ventricle-pulmonary artery.

Pulmonary artery stiffness (PAS) levels were higher in the PCOS group and PAS had a significantly positive correlation with HOMA-IR (r=0.545 and p<0.001) (Table 2 and Figure 1). Six patients (46%) with insulin resistance had higher PAS values than controls. The subgroup analysis of study patients who received treatment and those who received no treatment showed that pulmonary artery stiffness was higher in the non-treatment group (PAS= 5.15 ± 0.99 and 5.75 ± 1.02 respectively) but the difference was not statistically significant (p=0.084).

**Figure 1:** Correlation between HOMA-IR and PAS.

RV-PA coupling was impaired in 15 (34%) of the study group with mean levels 1.09 ± 0.23 and p value was significant between the two groups (p<0.001). Thirteen of these 15 patients were not receiving any treatment and the difference in terms of RV-PA coupling values between the subgroups, treated or nontreated, was also statistically significant. RV-PA coupling (mean ± SD) = 1.20 ± 0.22 for the treated group and 1.05 ± 0.22 for nontreated group. The p value was 0,048.

Kendall's tau b was 0.961 for PAS and 0.790 for RV-PA coupling.

## Discussion

It is well known that the risk of cardiovascular diseases is elevated in patients with PCOS due to increased insulin resistance and impaired glucose tolerance. Previously reported findings about insulin metabolism and resistance provide new clues in the treatment of PCOS and related complications.^11^

The clinical manifestations of insulin resistance are; HT, dyslipidemia and type 2 DM. Asymptomatic effects are endothelial dysfunction, procoagulant status, proinflammatory condition and smooth muscle cell proliferation. Ergun et al. found that patients with metabolic syndrome had higher aortic stiffness values than controls. The mechanism that shows how insulin resistance increases stiffness may be explained by its asymptomatic effects.^12^

Wang et al.^4^ reported in the CARDIA women's study that polycystic ovary syndrome is associated with higher left ventricular mass index and in another study, distinct abnormalities in both cardiovascular and metabolic features in PCOS were observed at an early age.^13^^,^^14^ These differences are reflected by an increased pulse pressure and a higher left ventricular end-diastolic but a lower tissue Doppler imaging of the right wall in systole. The results could indicate that women with PCOS already have subtle arterial dysfunction, which could lead to atherosclerosis in later life.

Pulmonary arterial stiffness and abnormal flow hemodynamics in pulmonary arterial hypertension are strongly associated with elevated right ventricular afterload and associated with disease severity and poor clinical outcomes in adults with PAH.^15^^–^^17^ RV-PA coupling can describe RV compensation in pulmonary hypertension and also in left-sided cardiac conditions and its importance has risen alongside increasing recognition of the pivotal role that RV plays in many cardiopulmonary conditions.^18^^–^^20^

We found that pulmonary artery stiffness, an indicator of pulmonary artery vasculature, was increased in PCOS and also associated with higher levels of HOMA-IR. RV-PA coupling, an indicator of pulmonary arterial compliance that has an important role in the pathogenesis of pulmonary arterial hypertension, is impaired in this patient group. This study is the first to evaluate pulmonary artery stiffness and RV-PA coupling in PCOS patients.

Considering all these complications and events, it has been shown in many previous studies and meta-analyses that the underlying pathology is insulin resistance. Although studies on the left ventricular and coronary artery disease are in the majority, pulmonary hypertension and right ventricular dysfunction have a significant place in mortality and establishes severe limitations for the patients’ quality of life. PCOS patients should be informed about cardiac risk and routine cardiac examinations should be recommended.

### Limitations

Our study had a few limitations. First, it was a single-center study with few participants. Another limitation of the current study was the short follow-up period. In addition, the evaluation of insulin resistance was only based on HOMA-IR. Further investigations with a longer duration and with larger groups are needed to assess the sustainability of the outcomes.

## Conclusion

In summary, this study is the first to provide preliminary data that PCOS patients have increased pulmonary artery stiffness and impaired RV-PA coupling.
